# Beyond viral load: Unravelling non-communicable disease patterns in Manicaland province, Zimbabwe

**DOI:** 10.4102/jphia.v16i1.587

**Published:** 2025-05-13

**Authors:** Kudzai F.V. Chokuona, Munyaradzi Mukuzunga, Tsitsi P. Juru, Addmore Chadambuka, Gerald Shambira, Notion T. Gombe, Mufuta Tshimanga

**Affiliations:** 1 Department of Primary Health Care Sciences, Faculty of Family Medicine, Global and Public Health Unit, University of Zimbabwe, Harare, Zimbabwe; 2 Manicaland Provincial Medical Directorate, Ministry of Health and Childcare, Mutare, Zimbabwe; 3 African Field Epidemiology Network, Harare, Zimbabwe

**Keywords:** non-communicable disease, human immunodeficiency virus, ART, Manicaland province, hypertension, diabetes mellitus

## Abstract

**Background:**

Non-communicable diseases (NCDs) among people living with human immunodeficient virus (HIV) are emerging and a leading cause of death in this population.

**Aim:**

To identify disease trends, prevalence and outcomes of NCDs among PLHIV.

**Setting:**

The study was conducted in Manicaland province.

**Methods:**

We reviewed secondary data from October 2013 to September 2023. Data on five priority NCDs were analysed: hypertension (HPT), diabetes mellitus (DM), chronic kidney injury (CKD), cancers and chronic respiratory conditions (CRC). Kaplan–Meier analysis and Cox proportional hazard analysis were performed, risk and hazard ratios reported at the 95% confidence level.

**Results:**

A total of *974* patient files were reviewed. The median age was *43* (*Q1 = 35; Q3 = 51*) years. A total of *409* (*42.0*%) were males and *565* (*58.0*%) were females. A total of *94* (*9.7*%) patients had HPT, *76* (*7.8*%) had DM, *6* (*0.6*%) had CKD, *9* (*0.9*%) had cancer and *3* (*0.3*%) had CRC. Controlling for age, gender and medication use, being on ART for more than 5 years and ageing were hazards to DM and HPT. Protease inhibitor-based regimen was a hazard to DM (hazard ratio [HR] *= 4.66, 95*% *CI: 2.54–8.54, p < 0.001*). Efavirenz-based regimen was protective in development of HPT (*HR = 0.47, 95*% *CI: 0.26–0.83*), *p* = 0.01.

**Conclusion:**

Hypertension and DM are the most common NCDs among people living with HIV. Prevalence of HPT and DM increased with age and duration on ART. To minimise complications related to NCD and HIV comorbidities, we recommend regular screening of NCDs at least monthly, and personalising treatment for hypertensive patients to efavirenz based regimens. We educated people living with HIV about the risks of NCDs and importance of healthy eating and regular exercise.

**Contribution:**

Integrated NCD and HIV care models.

## Introduction

People who are living with human immunodeficiency virus (HIV) have an increased risk of developing non-communicable diseases (NCDs).^1^ Non-communicable diseases associated with HIV and/or acquired immunodeficiency syndrome (AIDS) are emerging as the leading cause of death globally (equivalent to 74% of all deaths), with a doubled risk in the HIV-positive population than the non-AIDS related population.^2,3^ The gains in HIV care have led to an increase in older HIV patient cohorts associated with increased risk of NCDs.^4^ It is acceptable and feasible to integrate HIV and NCD services in resource limited settings.^5^ Annually, cardiovascular diseases, cancers, chronic respiratory diseases and diabetes mellitus (DM) account for *40*%, *23*%, *10*% *and 5*% of NCD-related global deaths, respectively.^3^

The risk of developing NCDs include HIV infection, the antiretroviral therapy (ART) regimens, ageing, physical inactivity, use of tobacco, air pollution, unhealthy diets, dyslipidaemia and alcohol abuse.^3,6^ People with more than one chronic condition have been lacking adequate integrated healthcare as some of these chronic conditions are missed during early stages, which is a major health system challenge.^7^

In low- and middle- income countries; the NCD burden accounts for *80*% of deaths with *30*% of these deaths occurring before reaching 60 years of age.^8^ According to the 2022 World Health Organization (WHO) updates, NCDs in Africa caused 100 000–400 000 deaths annually.^9^ Many people in resource limited settings cannot access care and treatment to NCDs.^10^ Furthermore, one in five people living with HIV have an NCD in low-income countries.^11^

Africa is facing the highest burden of DM, with Zimbabwe being among the countries with a high age standardised death rate of DM among females of *20–35 per 100 000* population.^4^ African countries also have a high prevalence of hypertension (HPT) ranging from *24*% *to 34*% among adults, with *82*% of them being neither aware nor on treatment.^4^ A review on monitoring progress of the African region in achieving national commitments towards NCDs has been slowest.^12^ In sub-Saharan Africa, the prevalent HIV associated NCDs include HPT, diabetes, cervical cancer, chronic respiratory diseases and metabolic syndrome.^13^

Zimbabwe has been experiencing a rise in NCDs in Africa.^14^ In another Zimbabwean study that used an individual multi-disease model evaluated that between 2015 and 2035, the most prevalent NCDs would be chronic kidney injury (CKD), HPT and cancer with an estimation of *59*% of people living with HIV having at least one NCD by 2035.^15^ Similarly, in Kenya, HPT and cancers were among the most prevalent NCDs among people living with HIV.^16^

At Victoria Chitepo Provincial Hospital (VCPH) an in-depth analysis of the burden of HIV associated NCDs has not been conducted. The burden of people living with HIV developing NCDs has been on the rise globally but this has not been evaluated in the cohort managed at VCPH, which is the referral facility for Manicaland province. We therefore analysed the data of NCDs among people living with HIV at VCPH from 2013 to 2023.

### Research question

What are factors associated with development of NCDs among people living with HIV at VCPH over the past 10 years?

### Objectives

- To analyse the demographic factors associated with development of NCDs among people living with HIV at VCPH from 2013 to 2023.
- To determine the clinical factors associated with the development of NCDs among people living with HIV at VCPH from 2013 to 2023.
- To evaluate the prevalence of NCDs among people living with HIV by ART regimen at VCPH from 2013 to 2023.
- To identify outcomes of HIV and/or NCDs comorbidity (alive, lost to follow-up, dead) among people living with HIV at VCPH from 2013 to 2023.
- To examine the trends of cumulative NCDs among people living with HIV at VCPH from 2013 to 2023.

## Research methods and design

### Study design

A retrospective cohort study of people living with HIV initiated on ART from October 2013 to September 2023, using secondary data was carried out.

### Study setting

The study was conducted at VCPH. Victoria Chitepo Provincial Hospital is situated in Mutare City 3.4 km north of Mutare town along the Mutare–Harare highway. Mutare is the third biggest city in Zimbabwe (after Harare and Bulawayo) and VCPH is the biggest government hospital in eastern Zimbabwe, a referral hospital for all the seven districts of the province. The hospital serves a population of 2 million people in the province. By December 2022, *132 755* people were on ART, in Manicaland province (District Health Information System 2 [DHIS2]).^17^

### Study population

Study participants included people living with HIV, enrolled on ART and records were maintained with case-based data entered into the Electronic Patient Management System (ePMS) and patient booklets at VCPH for the cohorts from 2013 to 2023.

### Sampling

All the *974* records with adequate patient data of those initiated on ART who presented at VCPH from October 2013 to September 2023 were enrolled into the study using ePMS and patient booklets.

### Data capture and analysis

Data which were captured from the ePMS and patient booklets, which includes the age, sex, follow-up status, cluster of differentiation 4 (CD4) count, ART regimen, ART start date, attendance records and the NCDs of the people living with HIV. The five major groups of NCDs were selected from the records among patients who developed NCDs after initiation on ART, which are HPT, DM, CKD, cancers and chronic respiratory conditions (CRC). Univariate analysis was performed through the calculation of proportions, means, medians and frequencies, using Epi Info 7 software. Kaplan–Meier analysis was performed using the same software, measuring against the event of interest, development of NCDs. The time to event was measured in months. Risk ratios and hazard ratios with 95% confidence intervals (CI) were generated and recorded from analysis. Cleaning of data was carried out before analysis. The Kaplan–Meier and log-rank tests were used for survival analysis of diabetic and hypertensive people living with HIV. Cox proportional hazards analysis was performed, adjusting for age, gender, and medication use, to identify significant factors associated with the hazard of developing an NCD.

### Ethical considerations

Permission to carry out the study was obtained from Manicaland Provincial Medical Directorate. Permission to review the patients’ records was obtained from the Medical Superintendent for VCPH. Confidentiality of the patients’ records and study participants was maintained. No names were included on the key informant guide.

## Results

After eliminating *42* records with missing data such as date of ART initiation, a total of *974* patient booklets were successfully reviewed. The demographic and clinical characteristics are shown in Table 1. Majority of the reviewed records constituted females: *565* (*58.0*%). Median age of the patients was *43* (*Q*1 *= 35; Q*3 *= 51*) years. Out of the *159* (*16.3*%) who developed NCDs, 59.1% of them had HPT.

**TABLE 1:** Demographic and clinical characteristics of human immunodeficient virus/non-communicable diseases cases, Manicaland province, Zimbabwe, October 2013 – September 2023.

Variable	Category	Frequency		Median
		*n*	%	
**Sex**	Male	409	42.0	-
	Female	565	58.0	-
**Case classification**	Hypertension	94	9.7	-
	Diabetes mellitus	76	7.8	-
	Cancers	9	0.9	-
	Chronic kidney disease	6	0.6	-
	Respiratory infections	3	0.3	-
**Outcome status**	Alive	864	88.7	-
	Lost to follow up	87	8.9	-
	Dead	23	2.4	-
**WHO stage**	Stage 1	400	41.2	-
	Stage 2	331	34.1	-
	Stage 3	217	22.3	-
	Stage 4	24	2.5	-
**Age (years)**	-	-	-	43
	Q_1_	-	-	35
	Q_3_	-	-	51
**Period of years on ART**	< 5 years	486	49.9	-
	≥ 5 years	488	50.1	-
**Type of current regimen**	Nevirapine (NVP) based	85	8.7	-
	Efavirenz (EFV) based	514	52.8	-
	Dolutegravir (DTG) based	917	94.2	-
	Protease inhibitor (PI) based	54	5.5	-

ART, antiretroviral therapy.

Majority of people living with HIV on the reviewed records, in this study had HPT *94* (*9.7*%) and DM *76* (*7.8*%). Out of the 974 reviewed records, *159* developed NCDs and the approximate prevalence rate was *120/10 000 per year* in Manicaland province. A total of *6* (*0.6*%) people living with HIV died from HPT and HIV comorbidity, *7* (*0.7*%) from DM and HIV comorbidity, 1 (0.1%) from cervical cancer and HIV comorbidity and *1* (*0.1*%) from CKD and HIV comorbidity in this cohort. A total of 15 NCD and/or HIV deaths out of the *159* people living with HIV who developed NCDs gave an approximate case fatality rate of *1000 per 10 000* people living with HIV with NCDs per year. The prevalence for having both HPT and DM increased from *1/10 000 per year* to *2/10 000 per year* to *8/10 000 per year* people living with HIV in 2020, 2021 and 2022, respectively.

In this study, people living with HIV who were 40 years or older had *1.10* (*95*% *CI: 1.07–1.14*) times risk of developing DM compared to those who were less than 40 years and it was statistically significant *p < 0.001*. Those who were initiated on dolutegravir (DTG) had *1.01* (*95*% *CI: 0.94–1.09*) times risk of developing DM compared to non-DTG users and it was not statistically significant *p = 0.82*. People living with HIV on WHO stage 3 or 4 (advanced HIV disease) had *2.72* (*95*% *CI: 1.95–3.93*) times risk of developing DM compared to those who were on WHO stage 1 or 2. This was statistically significant (*p < 0.001*).

**FIGURE 1:**
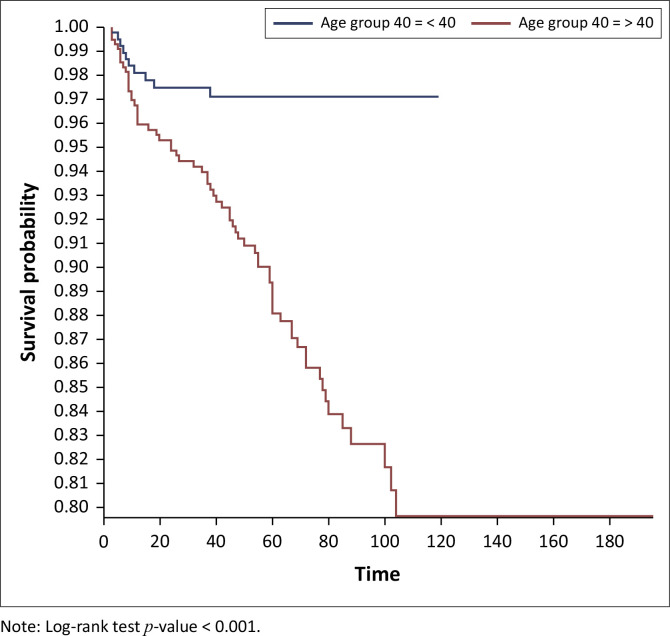
Kaplan–Meier survival analysis among people living with human immunodeficient virus who developed diabetes mellitus by age group, Manicaland province, Zimbabwe, October 2013 – September 2023.

**FIGURE 2:**
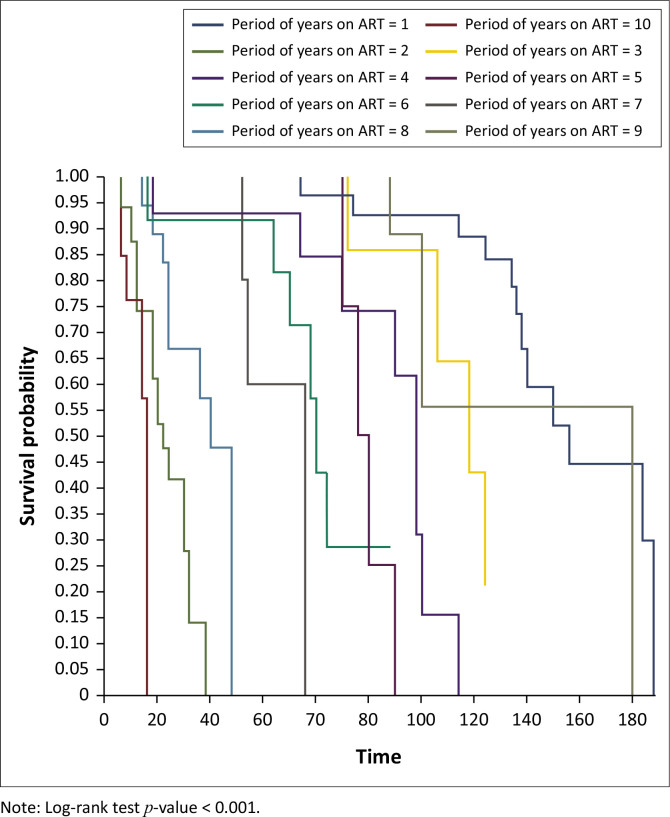
Kaplan–Meier survival analysis among people living with human immunodeficient virus who developed diabetes mellitus by duration on antiretroviral therapy, Manicaland province, Zimbabwe, October 2013 – September 2023.

**FIGURE 3:**
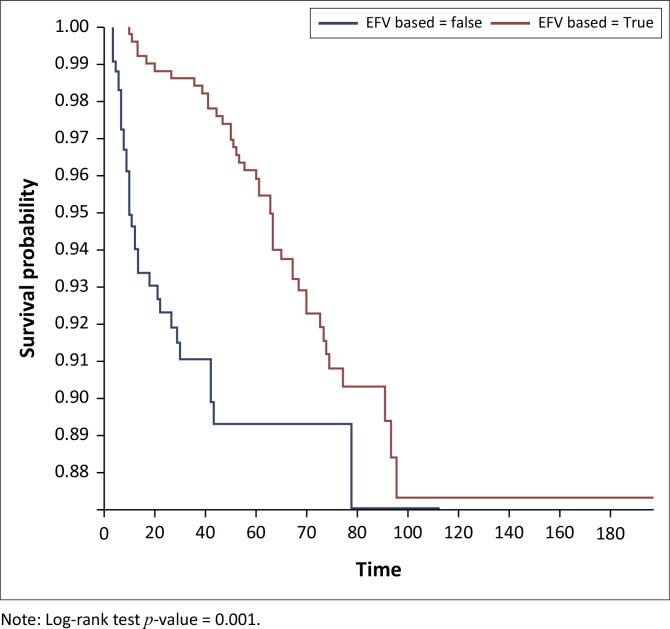
Kaplan–Meier survival analysis among people living with human immunodeficient virus who developed diabetes mellitus on efavirenz-based regimen, Manicaland province, Zimbabwe, October 2013 – September 2023.

**FIGURE 4:**
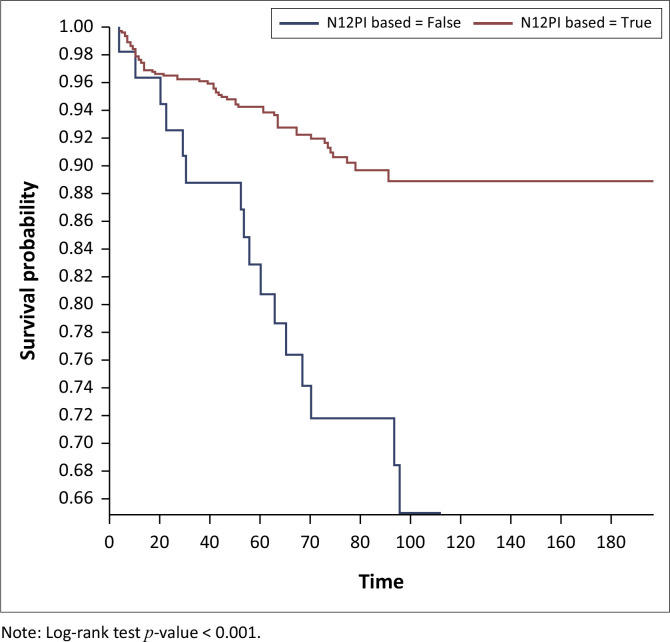
Kaplan–Meier survival analysis among people living with human immunodeficient virus who developed diabetes mellitus by protease inhibitors based regimen, Manicaland province, Zimbabwe, October 2013 – September 2023.

On Kaplan–Meier survival analysis, the probability of developing DM in people living with HIV at 40 years and above was higher than in those below 40 years and it was statistically significant (*p < 0.001*), Figure 1. The probability of developing DM increased with increasing years on ART and it was statistically significant (*p < 0.001*), Figure 2. The probability of developing DM was not different among DTG users and DTG non-users and it was not statistically significant (*p* = 0.43). The probability of developing DM was lower among EFV-based regimen users than EFV-based regimen non-users and it was statistically significant (*p = 0.001*); Figure 3. The probability of developing DM was lower among nevirapine (NVP)-based regimen users than NVP-based regimen non-users and but it was not statistically significant (*p = 0.20*). The probability of developing DM was higher among protease inhibitor (PI)-based regimen users than PI-based regimen non-users and but it was statistically significant (*p < 0.001*), Figure 4.

**FIGURE 5:**
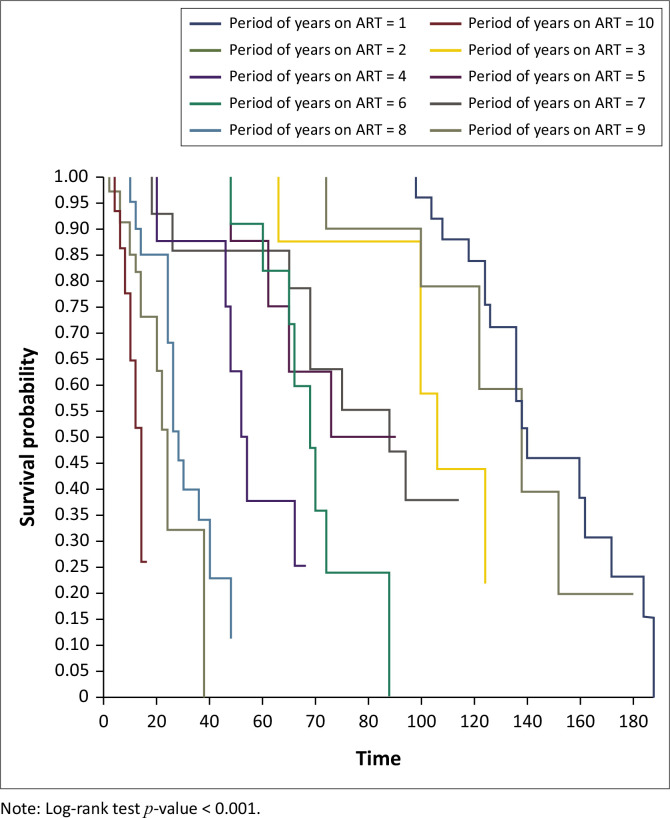
Kaplan–Meier survival analysis among people living with human immunodeficient virus who developed hypertension by duration on antiretroviral therapy, Manicaland province, Zimbabwe, October 2013 – September 2023.

**FIGURE 6:**
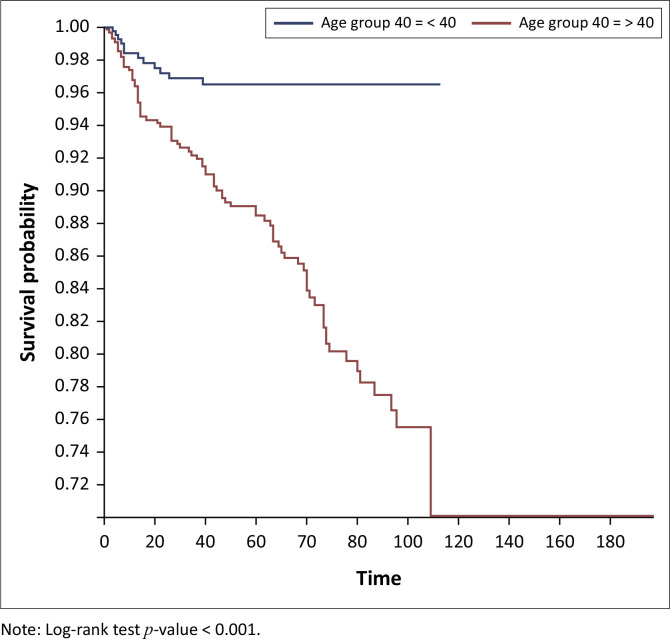
Kaplan–Meier survival analysis among people living with human immunodeficient virus who developed hypertension by age, Manicaland province, Zimbabwe, October 2018 – September 2023.

The probability of developing HPT among people living with HIV increased with increasing number of years on ART and it was statistically significant (*p < 0.001*); Figure 5. The probability of developing HPT at 40 years and above was higher than in those below 40 years and it was statistically significant (*p < 0.001*); Figure 6. The probability of developing HPT in people living with HIV was higher in females than males but it was not statistically significant (*p = 0.37*). There was no significant difference between the type of regimen being used and development of HPT.

**FIGURE 7:**
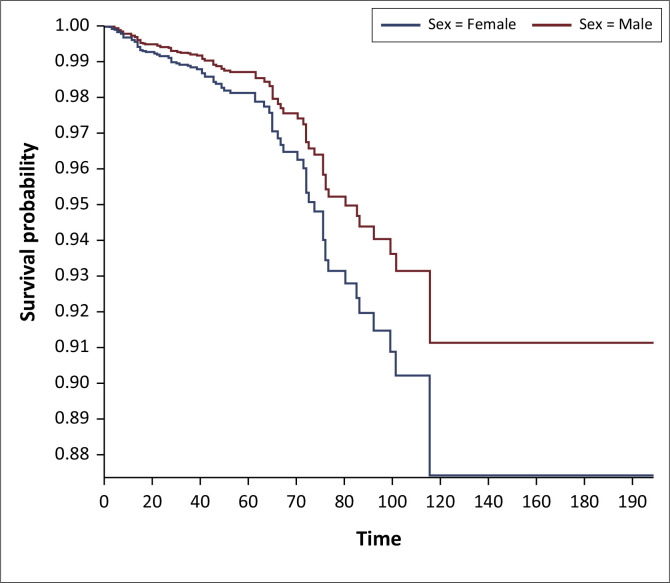
Cox proportional hazard analysis for development of hypertension, Manicaland province, Zimbabwe, 2013 – 2023.

Controlling for age, gender and medication use (Figure 7 and Table 2), the hazard rate for HPT was *5.99* (*95*% *CI: 2.78–12.91*) times higher in patients who had 5 or more years on ART compared to those who were on ART for less than 5 years and it was statistically significant (*p < 0.001*). Other hazards were being 40 years and above, *hazard risk (HR) = 4.78*, (*95*% *CI: 2.57–8.88*) and being diabetic, *HR = 4.63* (*95*% *CI: 2.71–7.91*). There was approximately a *53*% (*95*% *CI: 0.26–0.83*) lower hazard rate for HPT in the patients on efavirenz-based regimen compared to non-users of efavirenz-based regimen, *p = 0.01*.

**TABLE 2:** Cox proportional hazards for development of hypertension.

Variable	Hazard ratio	95% CI	*P*
ART period (≥ 5, < 5)	5.99	2.78–12.91	< 0.001*
Age group (years) (≥ 40, < 40)	4.78	2.57–8.88	< 0.001*
Diagnosed DM (yes, no)	4.63	2.71–7.91	< 0.001*
EFV based (yes, no)	0.47	0.26–0.83	0.01*
WHO stage (3 or 4, 1 or 2)	2.08	0.88–4.87	0.09
NVP based (yes, no)	0.92	0.42–2.01	0.34
PI based (yes, no)	0.69	0.30–1.58	0.38
DTG based (yes, no)	0.66	0.25–1.72	0.40
Sex (male, female)	0.69	0.45–1.09	0.09

NVP, nevirapine; PI, protease inhibitors; DTG, dolutegravir; EFV, efavirenz; ART, antiretroviral therapy; DM, diabetes mellitus; CI, confidence interval; WHO, World Health Organization.

* , Indicates statistical significance at *p* < 0.05.

**FIGURE 8:**
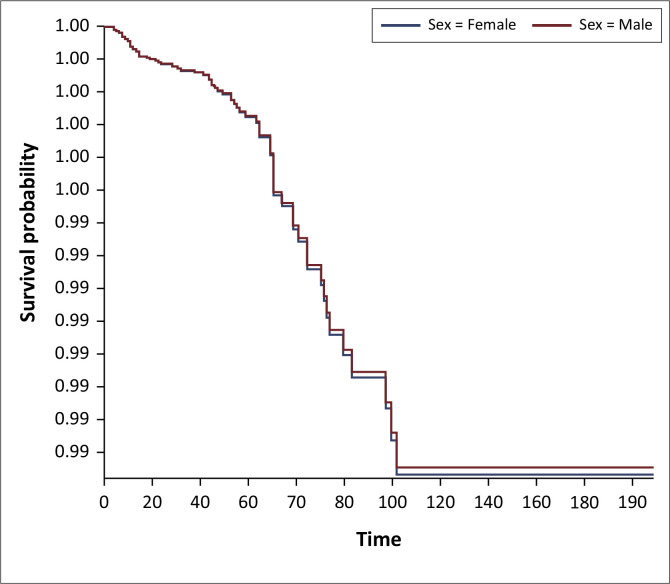
Cox proportional hazard analysis for development of diabetes mellitus, Manicaland province, Zimbabwe, 2013 – 2023.

The hazard rate for DM was *9.89*, (*95*% *CI: 4.53–21.59*) times higher in patients who had 5 or more years on ART compared to those who had less than 5 years and this was statistically significant, *p < 0.001*. The other hazards to development of DM were being on PI-based regimen, being greater than 40 years, being at WHO stage 3 or 4 and having HPT. The hazard rate for DM was lower among NVP based users, *HR = 0.93* (*95*% *CI: 0.37–2.34*) and efavirenz based regimen users, *HR = 1.03* (*95*% *CI: 0.57–1.88*), although statistically insignificant (*p = 0.87* and *p = 0.91*, respectively). However, there was no statistical significance between DTG use and development of DM and it was statistically insignificant, *HR = 1.70* (*95*% *CI: 0.57–5.03*), *p* = 0.33 (Figure 8 and Table 3).

**TABLE 3:** Cox proportional hazards for development of diabetes mellitus.

Variable	Hazard ratio	95% CI	*P*
ART period (≥ 5, < 5)	9.89	4.53–21.59	< 0.001*
PI based (yes, no)	4.66	2.54–8.54	< 0.001*
Age group (years) (≥ 40, < 40)	3.86	1.94–7.68	< 0.001*
WHO stage (3 or 4, 1 or 2)	3.75	1.75–8.07	< 0.001*
Diagnosed of HPT (yes, no)	3.52	2.06–6.02	< 0.001*
DTG (yes, no)	1.70	0.57–5.03	0.340
EFV based (yes, no)	1.03	0.57–1.88	0.910
NVP based (yes, no)	0.93	0.37–2.34	0.870
Sex (male, female)	0.98	0.62–1.56	0.940

NVP, nevirapine; PI, protease inhibitors; DTG, dolutegravir; EFV, efavirenz; HPT, hypertension; ART, antiretroviral therapy; CI, confidence interval; WHO, World Health Organization.

* , Indicates statistical significance at *p* < 0.05.

## Discussion

In our study, *one in six* people living with HIV on ART had an NCD. In Uganda *one in five* HIV-positive people had an NCD.^18^ The approximate similar prevalences in the study findings could be because of similar country policies aiming at integrating NCD services into HIV programmes.^19^ Moreover, the economic settings in Zimbabwe and Uganda are similar as both are low-resource settings. Low-resource settings are associated with poor living conditions, which have been reported to increase the risk of developing NCDs.^20,21^ In Zimbabwe, just like other sub-Saharan African countries, multimorbidity has been increasing among the adolescents and younger people with an increased risk of developing HIV-related NCDs.^22^

Among the reviewed records, the highest NCD among people living with HIV on ART in this cohort was HPT. This HPT results from prothrombotic changes and inflammation caused by HIV. The risk of HPT might be increased because of viral replication and viral translocation, stimulating immunological responses and metabolic disorders. These hypertensive effects have also been evidenced by Chastain et al.^23^

Ageing in people living with HIV on ART was directly associated with development of HPT as the exposure to HIV and various ART regimens also increased. As reduced immunity has been associated with ageing because of arterial stiffening, it is possible that inflammation from exposure to HIV and antiretroviral medicines increases the risk of HPT. Similarly, Chastain et al. and Daniel et al. evidenced that the time on ART, the various types of regimens and the duration of HIV diagnosis have been associated with HPT.^23,24^

The survival of people living with HIV with HPT and HIV comorbidity reduced with age as they deteriorated more over time with ageing and immunosuppression thereby shortening their lifespan. Similarly, in another study in Poland, the increase in duration with HIV disease and ageing has been directly associated with CVD risk.^25^ As people living with HIV were mostly screened on the yearly routine hospital visit, it delayed the diagnosis and treatment of HPT among other NCDs. Delayed diagnosis and treatment of HPT can be linked to the high morbidity and mortality among these people living with HIV as previously evidenced in an American study.^23^

A higher case fatality rate was evidenced in diabetics compared to other NCDs in this retrospective cohort study. This follows as management of both type 1 and type 2 DM can be critical as they both depend on the dietary and exercise lifestyle other than medication.^26^ The high number of deaths among the diabetics compared to other NCDs could be attributed to limited random blood sugar and glycosylated Hb screening among people living with HIV during routine visits thereby diagnosing diabetic patients at a chronic stage. Moreover, the risk of developing DM increased with age and years on ART from our study. Having more years on ART has been associated with increased risk of developing DM.^27^ The chronic HIV infection and exposure to ART regimens increases the diabetes rates.^27,28^ A previous study in Iran by Hadavandsiri et al. also supported the fact that glucose tolerance impairment increased with age.^29^

Tripathi et al. evidenced that exposure to ART and increasing years to various ART regimens was significantly associated with development of DM.^30^ On the contrary, people living with HIV on efavirenz or NVP in our study had higher chances of survival from developing DM compared to those on other regimens. This could be because of the lower influence on metabolic syndrome of non-nucleoside reverse transcriptase inhibitors (NNRTI) compared to other ART classes leading to a reduced risk of hyperglycaemia, dyslipidaemia and HPT, as previously evidenced by Nguyen et al.^31^

World Health Organization stage 3 or 4 signifies an advanced disease state that could result in chronic inflammation hence increasing the risk of developing DM in this cohort. Switching of regimens is thereby recommended while monitoring both the viral load and glucose metabolism.^23,32,33^ The potential risk of developing hyperglycaemia because of various ART switches and HIV metabolic syndrome reduced the survival of people living with HIV as evidenced in our study. This means that people living with HIV should not only be monitored for viral load but also for glucose metabolism and lipid metabolism. Contrary to our study findings, patients who were on WHO stage 1 had a higher risk of developing DM compared to other clinical stages in an Ethiopian study.^34^ However, in another study in Kenya, WHO staging had no significant association with the development of NCDs.^16^

Developing cervical cancer and any other cancers in HIV-positive individuals reduces the immunity as the body responds to the comorbidity. This immune suppression could have resulted in death of one of the people living with HIV in the study. We have observed a low number of cancer and NCD cases in this cohort and this has also been reported by Cheza et al. in Zimbabwe where cancers have the lowest incidence rate compared to other NCDs.^8^ The national early cervical cancer screening programmes and the human papilloma virus vaccine reduced the cancer incidence thereby reducing the cervical cancer and HIV related mortality.^35^

Our study had an HIV-positive patient who also suffered and died from chronic kidney disease. This could be attributed to poorly controlled HIV and exposure to other ART regimens, which results in a cytopathic effect on the glomerular filtration leading to renal failure as supported by Alfano et al., Naicker et al. and other researchers.^36,37^

Although our study reported the least number of people living with HIV with respiratory illnesses such as asthma, all of them were in WHO stage 3 in ART initiation, which is characterised by HIV and/or AIDS because of their weakened immunity.^38^ The use of ART has been evidenced to reduce respiratory infections and HIV related mortality.^39^

Dolutegravir has been hypothesised to cause insulin resistance through the chelation of its cofactor, the magnesium ions, thereby increasing the risk of hyperglycaemia. However, our study evidenced that there was no statistical significance between DTG use and development of DM as DTG was combined with other regimens giving a better cardio metabolic profile, with DTG associated with a high viral load suppression and the other regimens in the combination controlling metabolism. Moreover, our study constituted a 94% coverage of DTG users which could make the non-diabetic DTG users override the diabetic DTG users in analysis. Supporting evidence of using combined regimens to control metabolism has been reported by Tripathi et al.^30^

The protease inhibitor-based regimen was a hazard to DM in this study group. This follows as PI-based regimens produce enzymes that catalyse human proteins involved in homeostasis, metabolism and cell growth thereby inducing impaired glucose tolerance.^40^ This increases the risk of developing hyperglycaemia. Hughes et al. also evidenced that longer duration on PI increased the risk of developing DM.^41^

### Limitations

On updating the patient booklets, some of the files were disposed with information of interest such as previous ART history and patients who deceased five or more years ago, thereby eliminating some people living with HIV from the cohort study. The 42 records with missing information, the 87 lost to follow up patients and the 23 deaths could eliminate records with information of interest, which could contribute to some significant findings in the study.

## Conslusion and recommendations

Hypertension and DM are the common NCDs among people living with HIV because of the metabolic syndrome from HIV and the various ART regimens, causing high morbidity and mortality in this cohort. To minimise complications related to these NCD and HIV comorbidities, we recommend routine screening of NCDs on a monthly basis for early diagnosis and treatment. This can be improved by implementing screening programmes for prevalent NCDs such as HPT and DM. As a low-resource setting, we could seek donor funding not only for viral load testing but also for glycosylated Hb (HbA1c) screening for early diabetic management. We recommend HbA1c screening as it provides more reliable glucose levels over the past 2–3 months compared to random blood sugar, which detects daily sugar levels. The protective role of an efavirenz-based regimen is important in the management of hypertensive patients and so we recommend personalisation of treatment for hypertensive patients. Caution should be exercised when initiating PI-based regimens among people living with HIV who have existing metabolic disorders. Integrated care of both HIV and NCDs through routine viral load monitoring, blood pressure checks, glycosylated Hb testing, cancer screening and renal function tests, adjusting treatment accordingly, is recommended.
